# The Effects of Tormentic Acid and Extracts from *Callistemon citrinus* on *Candida albicans* and *Candida tropicalis* Growth and Inhibition of Ergosterol Biosynthesis in *Candida albicans*

**DOI:** 10.1155/2021/8856147

**Published:** 2021-09-21

**Authors:** Chido Bvumbi, Godloves Fru Chi, Marc Y. Stevens, Molly Mombeshora, Stanley Mukanganyama

**Affiliations:** ^1^Department of Biochemistry, Faculty of Science, University of Zimbabwe, Harare, Zimbabwe; ^2^Department of Organic Chemistry, University of Yaounde 1, P.O. Box 812, Yaoundé, Cameroon; ^3^Department of Medicinal Chemistry, Uppsala Biomedical Center, Uppsala University, P.O. Box 574, SE-751 23 Uppsala, Sweden

## Abstract

*Candida albicans* and *Candida tropicalis* are the leading causes of human fungal infections worldwide. There is an increase in resistance of *Candida* pathogens to existing antifungal drugs leading to a need to find new sources of antifungal agents. Tormentic acid has been isolated from different plants including *Callistemon citrinus* and has been found to possess antimicrobial properties, including antifungal activity. The study aimed to determine the effects of tormentic and extracts from *C. citrinus* on *C. albicans* and *C*. *tropicalis* and a possible mode of action. The extracts and tormentic acid were screened for antifungal activity using the broth microdilution method. The growth of both species was inhibited by the extracts, and *C. albicans* was more susceptible to the extract compared to *C*. *tropicalis*. The growth of *C*. *albicans* was inhibited by 80% at 100 *μ*g/ml of both the DCM: methanol extract and the ethanol: water extract. Tormentic acid reduced the growth of *C. albicans* by 72% at 100 *μ*g/ml. The effects of the extracts and tormentic acid on ergosterol content in *C. albicans* were determined using a UV/Vis scanning spectrophotometer. At concentrations of tormentic acid of 25 *μ*g/ml, 50 *μ*g/ml, 100 *μ*g/ml, and 200 *μ*g/ml, the content of ergosterol was decreased by 22%, 36%, 48%, and 78%, respectively. Similarly, the DCM: methanol extract at 100 *μ*g/ml and 200 *μ*g/ml decreased the content by 78% and 88%, respectively. A dose-dependent decrease in ergosterol content was observed in cells exposed to miconazole with a 25 *μ*g/ml concentration causing a 100% decrease in ergosterol content. Therefore, tormentic acid inhibits the synthesis of ergosterol in *C. albicans*. Modifications of the structure of tormentic acid to increase its antifungal potency may be explored in further studies.

## 1. Introduction

Fungal organisms of the genus *Candida* are the leading cause of yeast infections among humans. More than 17 *Candida* species are known to cause infection in human beings. *Candida albicans* is the most virulent and most predominant, causing about 90% of all invasive yeast infections followed by *Candida tropicalis* that causes about 7% of all yeast infections [1]. Infection by fungal pathogens of the genus *Candida* is commonly known as Candidiasis. Candidiasis commonly presents itself as mucocutaneous candidiasis in which lesions on the skin can be seen. Vaginal candidiasis results from the invasion of vaginal walls. Candidiasis can also affect the mouth, a condition commonly known as oral thrush [2]. Fungal infections can lead to dysphagia, which is characterized by pain during swallowing due to infection of the esophagus. The proliferation of *Candida* can have catastrophic effects if the pathogenic fungi reach vital organs [3].

*Candida* species are opportunistic pathogens that cause candidiasis in humans [4]. These organisms are referred to as opportunist pathogens as they constitute part of normal flora and generally do not cause any harm to immune-competent individuals. For immune-compromised individuals, however, infection by *C*. *albicans* and *C*. *tropicalis* among other pathogens can cause serious illness. In some cases, infection by these pathogens can even be fatal. Some of the predisposing factors for candidiasis include HIV and tuberculosis infection, cancer, diabetes, and the use of immune-suppressing drugs [5]. With the increasing cases of diseases, such as HIV, the incidence of invasive candidiasis has escalated over the past decades. The progression of *Candida* infection is dependent not only on the virulence factors but also on the host immune response [6]. Drugs have been formulated to treat *Candida* infections. These drugs include azoles, polyenes, and allylamines [7]. Many of these available drugs target the ergosterol biosynthesis pathway that is unique to fungal membranes. Unfortunately, over the past decade, there has been a growing resistance of *Candida* species to these available drugs. It has become of importance to investigate other potential sources of antifungal agents. Plants can act as good sources of therapeutic phytochemicals [8].

Ergosterol is the major sterol found in fungal membranes. It was first isolated in 1889 by Charles Tanret from ergot [9]. Some of the physiological functions of ergosterol include maintaining membrane integrity and fluidity [10]. Fungal cells also require ergosterol for the completion of their cell cycle, a process known as the “sparkling function” [11]. In fungal cells, ergosterol also plays a role in mating. A study by Aguilar et al. [12] showed that ergosterol had an effect on the shape of the cell and that disruption of the cell figure affected the fusion of fungal cells during mating. The importance of this pathway is as a target for antifungal drugs is the fact that the biosynthesis of ergosterol occurs exclusively in fungal membranes.

Natural plant products are documented as rich sources of several compounds that may serve as scaffolds for the development of drugs with anti-*Candida* action [8, 13]. Studies have shown that *Callistemon citrinus* has antimicrobial effects [14–16]. Several phytochemicals have been isolated from the plant including the pentacyclic triterpene and tormentic acid [17]. Tormentic acid is a triterpene found in a variety of plants. Though not extensively studied, some reports have been made about the anticancer and antimicrobial nature of this phytochemical [18, 19]. Phytochemical studies on *C*. *citrinus* seem to also suggest that terpenoids including tormentic acid are responsible for the antifungal activity exhibited by its extracts [17]. To the best of our knowledge, no studies have been done to investigate the effect of tormentic acid on ergosterol biosynthesis. The study aimed to initially determine the effects of tormentic acid and extracts from *C*. *citrinus* on the growth *C*. *albicans* and *C*. *tropicalis* and to determine its effects on the synthesis of ergosterol in *C*. *albicans*.

## 2. Materials and Methods

### 2.1. Reagents and Materials

All chemical reagents used in this study were purchased from Sigma-Aldrich (Darmstadt, Germany). Solvent reagents used were dichloromethane (DCM), methanol, ethanol, dimethyl sulphoxide, (DMSO) and hexane. Other reagents used were Sabouraud dextrose broth (SDB), Sabouraud glucose 2% agar (SGA), 3-(4,5-dimethylthiazolyl)-2,5-diphenyltetrazolium bromide (MTT), and potassium hydroxide (KOH). The drugs miconazole and fluconazole were also obtained from Sigma-Aldrich (Steinheim, Germany). *Candida albicans* (NCPF 3255) lab strain was purchased from Sigma-Aldrich (Steinheim, Germany). *Candida tropicalis* clinical strain was obtained from the Department of Medical Microbiology at Parirenyatwa Hospital in Harare, Zimbabwe.

### 2.2. Plant Collection and Preparation

*Callistemon citrinus* leaves were collected from the University of Zimbabwe, Department of Biochemistry, Harare, Zimbabwe, latitude −17.7840, longitude 31.0530. The plant identity was authenticated and classified by Mr. Christopher Chapano, a taxonomist at the National Herbarium and Botanic Gardens (Harare, Zimbabwe). Leaves were separated and dried at a temperature of 50°C in an oven (Memmert, Schwabach, SRG, Germany) for three days. The dried leaves were ground to obtain a powder using a two-speed blender (Cole Parmer Instrument Company, Connecticut, USA), to optimise the solvent contact during the extraction. To each of the two plastic beakers, 50 g of the leaf powder was added. A 50 : 50 v/v solution of ethanol : water solvent solution was added to one beaker and the other, a 50 : 50 (v/v) solvent solution of dichloromethane (DCM) and methanol, was added. The two beakers were covered with foil paper and left to stand for 24 hours at room temperature with occasional swirling. Two filter papers (12.5 cm diameter, Sharkskin, 10347511, USA) were placed in two separate funnels and through one the ethanol: water extract was further filtered and to the other the DCM: methanol extract in solvent was filtered. These were filtered into two well-labeled beakers. The two samples were left to dry for 10 days under a stream of air from a fan.

### 2.3. Isolation of Tormentic Acid Using Column Chromatography

The extract was run on a column with 100% hexane initially as the eluting solvent. Batch gradient system column elution was employed starting with a solvent of low polarity to that with high polarity. The batch gradient system was a 20-step gradient elution with a gradual increase of polarity. Polarity on the column was increased by introducing a polar solvent, to hexane up to a solvent system with 100% ethyl acetate. Methanol was added to 100% EA up to 90% EA and 10% methanol. Fractions of 250 ml were collected and concentrated using a Buchi RII rotary evaporator (Buchi R2, BÜCHI Labortechnik AG, Flawil, Switzerland). Thin layer chromatography was used for the analysis of the collected fractions. The fractions, which came out having the same Rf values, were pooled. The pooled fractions were left to evaporate to dryness for crystals to form. Purification of the pooled fractions was performed by washing away the impurities. Single spots observed on the developed chromatograms under UV (354 and 365 nm) after staining with sulphuric acid were deemed pure and were subjected to NMR/MS analyses.

### 2.4. NMR Analyses and Determination of the Mass of the Isolated Compound

**Figure 1:**
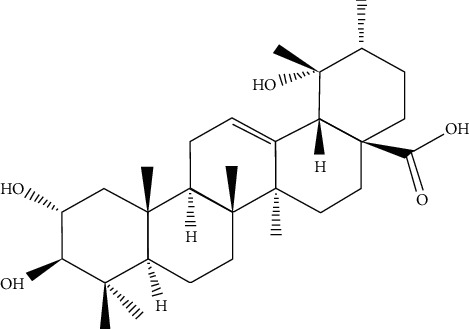
Structural representation of tormentic acid, a compound isolated from *C. citrinus*.

^1^H NMR spectra were recorded at 400 MHz and ^13^C NMR spectra at 100 MHz. The chemical shifts for ^1^H NMR and ^13^C NMR were referenced to TMS via residual solvent signals (^1^H, CDCl_3_ at 7.26 ppm; ^13^C, CDCl3 at 77.36 ppm; ^1^H, DMSO*-d*_6_ at 2.45 ppm; ^13^C, DMSO*-d*_6_ at 39.43 ppm, ^1^H, CD_3_OD at 3.31 ppm; ^13^C, CD_3_OD at 49.0 ppm). 2D NMR experiments were run using standard pulse sequences. Molecular formulae were determined by electrospray ionisation with a 7-T hybrid ion trap and a TOF detector running in positive or negative mode. One of the isolates was identified as tormentic acid based on NMR data and similar data in the literature (Figure 1).

### 2.5. Preparation of Fungal Cultures

*Candida albicans* NCPF 3255 (Sigma-Aldrich, Fancy Road Poole, Dorset UK) and *Candida tropicalis* cells were resuscitated in Sabouraud dextrose broth (SDB). In a disinfected Labotec Biosafety cabinet (Scientific Engineering Pvt. Ltd., Industrial North, South Africa). After 24 hours, the cells were subcultured on solid Sabouraud dextrose agar and incubated for 24 hours at 37°C in the Lab Companion Incubator SI 300 (Jeio Tech Co. Ltd., Seoul, Korea). Plates were stored in a refrigerator at 4°C and renewed every 3 weeks. The same procedure for the resuscitation of fungal cells was done for *C. tropicalis*.

### 2.6. Screening for Antifungal Effects of the Extract and Tormentic Acid

Three different colonies from the agar medium were grown in SDB. The cell concentrations were standardised using 0.5 M MacFarland solution to 2 × 10^8^ c.f.u/ml according to protocol by Ramirez-Arcos [19]. The broth microdilution assay was conducted to determine the effect of the two crude extracts and pure tormentic acid on both *C*. *albicans* and *C*. *tropicalis* according to Wiegand et al. [20]. Double dilution concentrations of 0 *μ*g/ml to 100 *μ*g/ml of the extract and tormentic acid were prepared in SDB. Fluconazole or miconazole was used as the positive control. Wells containing either the extract only or SDB only served as controls. The 3-(4,5-dimethythiazol-2-yl)-2,5-diphenyl tetrazolium bromide (MTT) assay was done to investigate the presence/absence of viable cells in all wells after incubation according to [21]. A volume of 20 *μ*l of the MTT solution was added to each of the 96 wells on the microtitre plates. The plates were incubated at 37°C for at least 2 hours. Color changes were quantified at 590 nm using a microplate reader (Genios Pro, Tecan Group Ltd., Grödig, Austria). The Minimum inhibitory concentrations (MIC) for the extract or tormentic acid were recorded. Graphs were plotted to show cell viability using GraphPad Prism6 (Version 6.0 GraphPad Software Inc., San Diego, California, USA).

### 2.7. Determination of Ergosterol after Exposure to Extracts and Tormentic Acid

Determination of the amount of ergosterol was done according to the method by Arthington-Skaggs et al. [22] with slight modifications. *Candida* cells were grown on Sabouraud dextrose agar (SDA). The cells were subcultured in 20 ml of SDB and incubated for 24 hours at 37°C in a Mini Incubated Shaker (Lab Doctor™, MidSci Co, St. Louis, North America). The working solutions for DCM: methanol extract and for tormentic acid were 200 *μ*g/ml, 100 *μ*g/ml, 50 *μ*g/ml, and 25 *μ*g/ml. The working concentrations for miconazole (positive control) were 25 *μ*g/ml, 12.5 *μ*g/ml, 6.3 *μ*g/ml, and 3.1 *μ*g/ml. The negative control had the cell culture and media. These solutions were incubated for 24 hours at 37°C with shaking at 170 rpm. After incubation, the solutions were centrifuged at 2 700 rpm for 5 minutes in a Rotofix centrifuge (Taufkirchen, Germany). The cells were washed with distilled water and the net weight of the cells was recorded. A volume of 3 ml 25% alcoholic potassium hydroxide was added to each of the *Candida* pellets and mixed for one minute. The cell suspensions were then transferred to clean borosilicate glass tubes and incubated at 85°C for an hour. The cells were left to cool at room temperature. A solvent mixture of sterile water and *n*-hexane (1 : 3 v/v) was added to each of the cell suspensions. The mixture was vortexed for 3 minutes. The clear hexane layer was drawn out and transferred to a clean tube and stored at −20°C. The hexane extract was diluted in ethanol (1 : 4 v/v) and scanned at 220 nm and 300 nm using a UV/VIS spectrophotometer (Biobase BK D560, Jinan, Shandong, China). The amount of ergosterol was calculated as the difference in absorbance between the 230 nm and the 281.5 nm peak per unit mass of the fungal cell pellet using the following equation:

(1) $$\begin{array}{c} \text{ergosterol amount}=\frac{\left[\text{F}.\left(\text{A}281\text{.}5/290\right)-\text{F}.\left(\text{A}230/518\right)\right]}{\text{pellet mass}}, \end{array}$$

where A_281.5_ is the absorbance of hexane extract at 281.5 nm, A_230_ is absorbance between 230 nm and 281.5 nm, F is the dilution factor of hexane extract in ethanol, and 290 and 518 are extinction values determined for crystalline ergosterol.

### 2.8. Statistical Analyses

All data in this study were analysed using GraphPad prism software, versions 7 and 8 (GraphPad Software Inc., San Diego, California, USA). Statistical analysis of data was done using one-way analysis of variance (one-way ANOVA) followed by Dunnett's test for multiple comparisons. Tests for antifungal susceptibility of *Candida* to crude extracts and miconazole were done in quadruplicate. Determination of ergosterol content was done in two separate experiments and mean values were compared to the negative control with cells only. Means were considered significant when the *p* value was less than 0.05.

## 3. Results

### 3.1. Sample Preparation and Yields of Extraction of the Crude Extract

The mass of the extracts obtained from 50 g of leaf extract in 100 ml of solvent solution was weighed after drying. The ethanol: water extract and the DCM: methanol crude extract yields 4.8 g (9.2%) and 1.6 g (3.2%) of powdered extract, respectively.

### 3.2. Isolation and Identification of Tormentic Acid

Tormentic acid: 2*α*, 3*α*,19*α*-trihydroxyurs-12-en-28-oic acid [23, 24] was isolated from the leaf extracts of *C*. *citrinus*.

The following spectral characteristics were obtained: white powder; ^1^H-NMR (DMSO, 400 MHz) *δ* (ppm): 5.14 (1H, *br* s, H-12), 3.49 (1H, m), 2.74 (1H, d, *J* = 9.2, H-3), 2.12 (1H, d, *J* = 11.2, H-18), 2.00–1.20 (CH_2_ and CH region), 1.04 (3H, s, H-27), 0.93 (3H, s, H-23), 0.93 (3H, s, H-25), 0.92 (3H, s, H-30), 0.83 (3H, d, *J* = 6.4, H-29), 0.77 (1H, t, H-5), 0.75 (3H, s, H-26), 0.72 (3H, s, H-24). ^13^C-NMR (DMSO, 100 MHz) *δ* (ppm); 178.7 (C-28), 138.7 (C-13), 124.9 (C-12), 82.7 (C-3), 70.2 (C-20), 67.6 (C-2), 55.2 (C-5), 52.8 (C-18), 47.5 (C-17), 47.5 (C-1), 47.4 (C-9), 46.4 (C-14), 40.5 (C-10), 39.4 (C-8), 39.3 (C-4), 38.9 (C-21), 38.8 (C-19), 36.8 (C-22), 33.0 (C-7), 30.6 (C-16), 29.3 (C-23), 27.9 (C-15), 23.7 (C-27), 23.4 (C-11), 21.5 (C-30), 18.6 (C-6), 17.6 (C-24), 17.5 (C-26), 17.4 (C-24), 16.9 (C-29), 16.9 (C-25). Similar spectral characteristics have been reported in [25].

**Figure 2:**
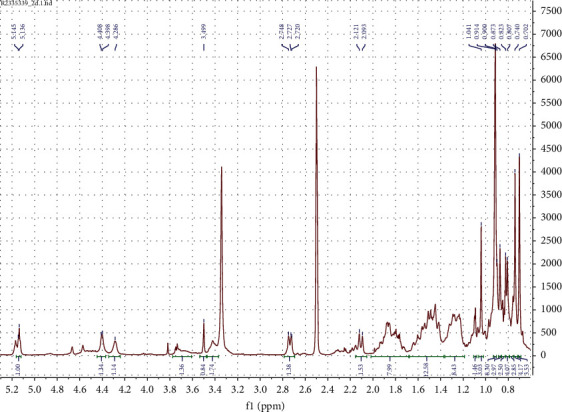
^1^H NMR (DMSO, 400 MHz) spectrum of tormentic acid.

**Figure 3:**
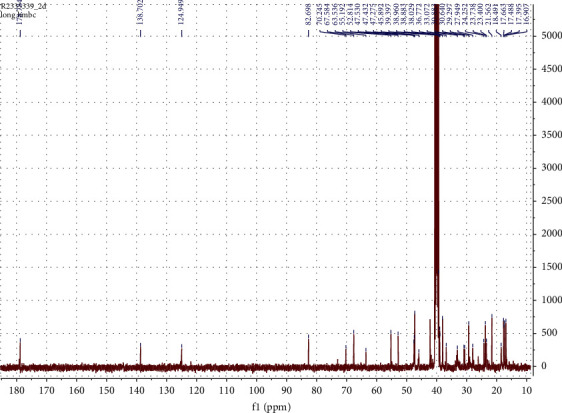
^13^C NMR (DMSO, 100 MHz) spectrum of tormentic acid.

**Figure 4:**
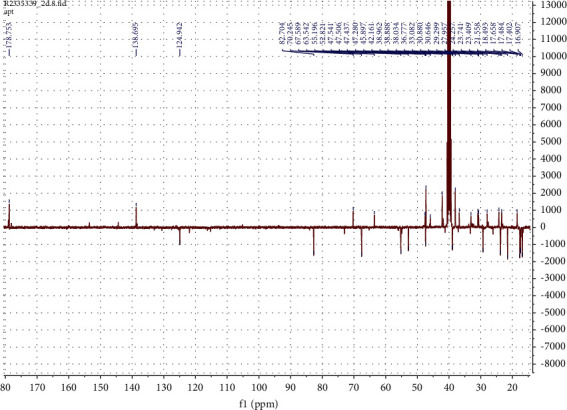
^13^C APT-NMR (DMSO, 100 MHz) spectrum of tormentic acid.

NMR spectra are shown in Figures 2–4.

### 3.3. Screening for Antifungal Effects of the Extract and Tormentic Acid on *C. albicans*

**Figure 5:**
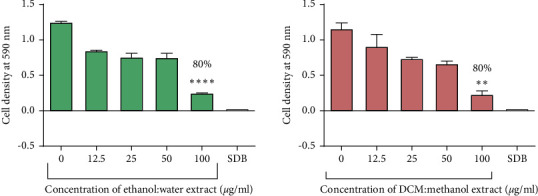
The effect extracts from *C. citrinus* on *C. albicans*. The graph shows a dose-dependent decrease in *C. albicans* cell density as the concentration of the ethanol: water extract and the DCM: methanol increased. Percentage inhibition of 80% was observed at 100 *μ*g/ml. The data represent a mean of four replicates. These data were analysed using one-way ANOVA followed by Dunnett's test for multiple comparisons, comparing means to the negative control (SDB). Error bars indicate standard deviation; ^*∗∗∗∗*^*p* < 0.00001.

There was a general decrease in cell density with an increase in ethanol: water extract concentration and the DCM: methanol extract concentration from 0 *μ*g/ml to 100 *μ*g/ml for both species of *Candida* (Figure 5).

**Figure 6:**
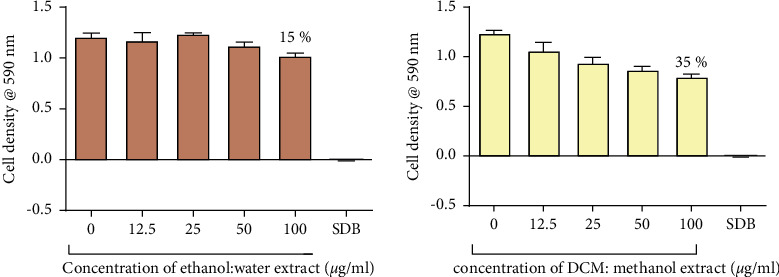
The effect extracts from *C. citrinus* on *C. tropicalis*. Percentage inhibition of 15% and 35% was observed at 100 *μ*g/ml of the ethanol: water extract and DCM: methanol extract, respectively. The data shown in this graph represent the mean for four replicates. These data were analysed using one-way ANOVA followed by Dunnett's test for multiple comparisons, comparing means to the negative control (SDB).

**Figure 7:**
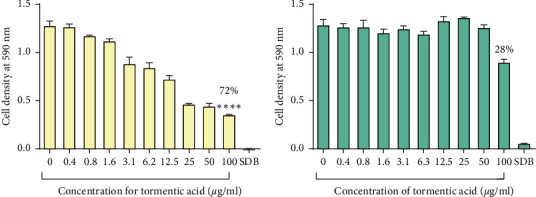
The effect of tormentic acid the growth of *C. albicans* (a) and *C. tropicalis* (b). The graph shows a dose-dependent decrease in cell density as the concentration of tormentic acid was increased. Percentage inhibition of 72% and 28% was observed at 100 *μ*g/ml for *C. albicans* and *C. tropicalis,* respectively. These data represent a mean of four replicates. The data were analysed by one-way ANOVA and Dunnett's test for multiple comparisons. Mean values were statistically compared to the negative control (SDB). Error bars indicate standard deviation; ^*∗∗∗∗*^*p* < 0.00001.

The highest cell density was recorded in cells with the control that contained cells with no exposure to any test compound. The lowest cell density was recorded in wells containing 100 *μ*g/ml of the ethanol: water extract and the DCM: methanol extract with a percentage inhibition of 80%. The ethanol: water extract and the DCM: methanol extracts reduced the growth of *C*. *tropicalis* by 15% and 35%, respectively, at 100 *μ*g/ml (Figure 6). There was no growth of cells observed in the SDB media only wells. Tormentic acid reduced the growth of *C. albicans* and *C. tropicalis* by 72% and 28%, respectively, at 100 *μ*g/ml (Figure 7). Miconazole significantly reduced the growth of both species with MICs of 1.6 *μ*g/ml and 12.5 *μ*g/ml for *C. albicans* and *C. tropicalis,* respectively.

### 3.4. Ergosterol Quantification after Exposure to Extracts and Tormentic Acid

**Figure 8:**
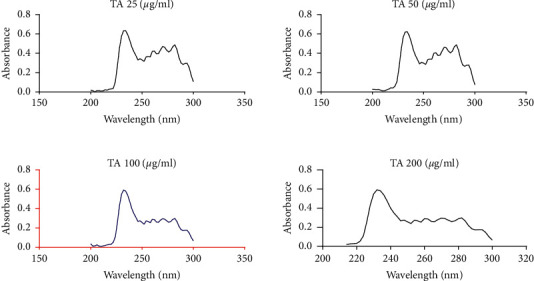
Absorbance spectra of ergosterol from *C. albicans* cells exposed to tormentic acid. Cell pellets of *C. albicans* were exposed to 25 *μ*g/ml, 50 *μ*g/ml, 100 *μ*g/ml, and 200 *μ*g/ml of tormentic acid (TA). The graphs show the UV/Vis scan of the extracted sterols from 200 nm to 300 nm. There was a similar spectra pattern, and a general decrease with increased concentration of tormentic acid was observed.

**Figure 9:**
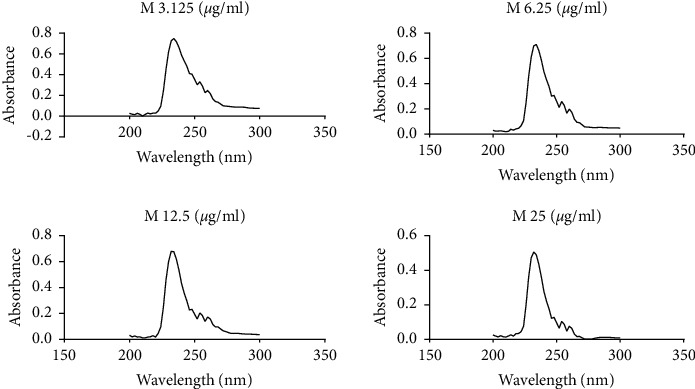
Absorbance spectra of ergosterol from *C. albicans* cells exposed to miconazole. Cell pellet was exposed to miconazole concentrations of 3.125 *μ*g/ml, 6.25 *μ*g/ml, 12.5 *μ*g/ml, and 25 *μ*g/ml of miconazole (M). The graphs show the UV/Vis scan from 200 nm to 300 nm.

Using a UV/Vis scanning spectrophotometer (Biobase, Jinan, Shandong, China), sterols extracted with hexane were scanned within the wavelength range of 200 nm to 300 nm. The absorption spectra for ergosterol extracted from cells exposed to varying concentrations of tormentic acid and miconazole are shown in Figures 8 and 9 , respectively.

**Figure 10:**
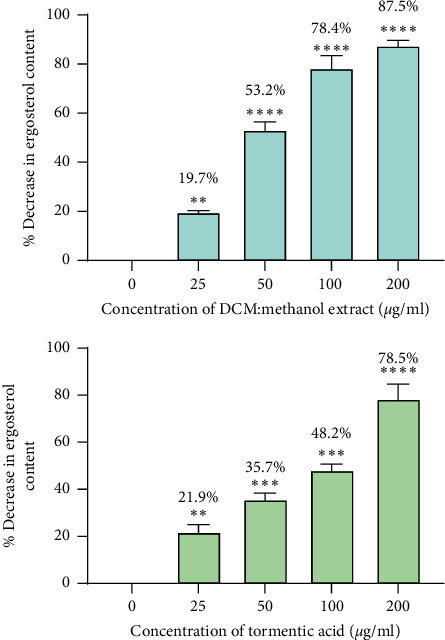
Percentage decrease in ergosterol with increasing concentration of DCM: methanol extract and tormentic acid. The data represent the mean of two experiments, and the standard deviation was shown by the error bars. ^*∗∗∗∗*^*p* < 0.00001 and ^*∗∗*^*p* < 0.01.

**Figure 11:**
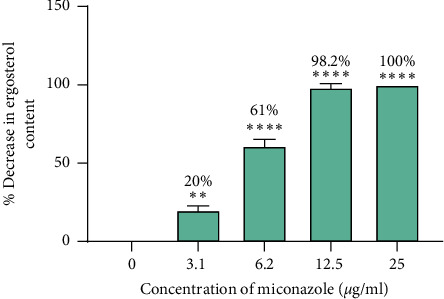
The effects of miconazole on cells exposed to miconazole. Ergosterol content decreased as the concentration of miconazole was increased. A percentage decrease in ergosterol of 100% was observed at 25 *μ*g/ml of miconazole. Data were analysed by one-way ANOVA. The data represent the mean of two experiments, and the standard deviation was shown by the error bars. ^*∗∗∗∗*^*p* < 0.0001 and ^*∗∗*^*p* < 0.01.

Percentage decreases in ergosterol were calculated relative to ergosterol content in unexposed cells. These data are summarised in Figure 10 for the cells exposed to the DCM: methanol extract and tormentic acid. The percentage decrease in ergosterol for cells exposed to miconazole is shown in Figure 11. The highest concentration of DCM: methanol extract (200 *μ*g/ml), tormentic acid (200 *μ*g/ml), and miconazole (25 *μ*g/ml) gave 87.5%, 78.5%, and 100% inhibition of ergosterol content, respectively.

## 4. Discussion

The therapeutic properties of *C*. *citrinus* are attributed to the presence of active phytochemicals found in the plant leaves, flowers, stems, bark, and roots [26]. The amount and composition of phytochemicals depend on the plant part, from which the phytochemicals are obtained [27, 28]. Extraction of phytochemicals, in this study, was done from the leaves of the *C*. *citrinus*. Antifungal susceptibility tests on *C. albicans* showed significant inhibition of fungal growth by *C. citrinus* extracts. The percentage inhibition at the highest concentration investigated was compared for all treatments with the cells only readings being used as reference points. *C. albicans* was more susceptible to tormentic acid and the two crude extracts than *C. tropicalis*. The DCM: methanol extract generally showed higher antifungal effects on both *C. albicans* and *C. tropicalis* when compared to tormentic acid and the water: ethanol extract. For tests on *C. tropicalis*, however, generally lower levels of cell growth inhibition were observed compared to *C*. *albicans*. *C. albicans* was, therefore, more susceptible and the DCM: methanol extract was the most potent antifungal extract, and for this reason, further studies were carried out using *C*. *albicans* only. Several mechanisms may attribute to the effect brought about by natural products. These include alteration of the membrane permeability, disruption of biological processes that are important for the microorganism, like the synthesis of ergosterol an essential component of the fungal membrane [29], disruption of the growth mechanism of microbe, and alterations in the respiratory chain [30].

The extracts generally showed higher antifungal activity than tormentic acid. This may have been due to the synergistic effects of phytochemicals within the crude extracts. The DCM: methanol extract showed more potency against the fungal strains. This may be due to higher concentrations of some specific antifungal biomolecules within this extract as the DCM: methanol extract can extract nonpolar and polar substituents from *C. citrinus* leaves. The nonpolar active secondary metabolites including tormentic acid or their combined activity with other polar phytochemicals may be responsible for the relatively higher antifungal activity in the ethanol: water extract [31]. Other authors have been able to isolate sterols and terpenoids from *C. citrinus* leaves, which showed significant inhibitory effects against *Candida* species. Through GC-MS analysis, Srivastava et al. [32] and Salem et al. [33] have identified the presence of high amounts of essential oils, such ɑ-pinene, limonene, 1.8 cineole, and *α*-terpineol, as well as high terpene content in nonpolar extracts of *C. citrinus*. Kim et al. [34] studied the antifungal effects of the methanolic extracts on *Aspergillus niger*. Their results showed that these essential oils as well as terpenoids had fungistatic effects. Cock [35], however, reported that the methanolic extracts of *C. citrinus* leaves had a low antifungal effect against *C*. *albicans* and no significant effect against *Aspergillus niger*. The antifungal activity observed with the DCM: methanol extract in this current study may be linked to the high yield of the extract obtained using a combined solvent solution. Comparison of antifungal susceptibility is difficult as different researchers use different methods including disc diffusion and broth dilution methods [20].

*C*. *tropicalis* was found to be less susceptible to the extracts and tormentic acid. It has also been documented to be resistant to the first line azole fluconazole. Various studies have been carried out to determine the underlying cause of the lack of susceptibility of *C. tropicalis* strains to antifungal agents. One of the extensively studied areas is the change in the azole target that is the enzyme lanosterol C14*α* demethylase or CYP 51. These studies have shown mutations in the amino acid sequence of CYP 51 enzyme. Some of the most common mutations discovered were in Y132F, S154L, and K143R amino acid residues, which are found within the active site of the enzyme [36, 37]. This change in amino acid sequence could result in reduced binding capacity of azole drugs and even other antifungal agents, such as the tested tormentic acid as well as extracts.

*C*. *albicans* was shown to be more susceptible to *C. citrinus* extracts when compared to *C*. *tropicalis*, and, therefore, it was used in the subsequent ergosterol biosynthesis investigation assay. The cells were treated with varying concentrations of tormentic acid, and ergosterol production was monitored. The effect of DCM: methanol extract that showed the highest antifungal effect on *C. albicans* was also investigated. The results showed that the concentration of extracts was directly proportional to the amount of ergosterol per unit mass of *C. albicans* cells. For concentrations of 25 *μ*g/ml, 50 *μ*g/ml, 100 *μ*g/ml, and 200 *μ*g/ml of tormentic acid, the percentage decrease in ergosterol was 21.9%, 35.7%, 48.2%, and 78.5%, respectively. The same concentrations of DCM: methanol extract gave a percentage decrease of 19.7%, 53.2%, 78.4%, and 87.5%. The percentage decrease in ergosterol content was relatively higher than tormentic acid with DCM: methanol treatment. The effect of miconazole on ergosterol biosynthesis in *C. albicans* was determined at concentrations 3.125 *μ*g/ml, 6.25 *μ*g/ml, 12.5 *μ*g/ml, and 25 *μ*g/ml. The percentage decreases in these tests were 20.0%, 61%, 98.2%, and 100%, respectively. Ergosterol synthesis in miconazole treated cells was almost completely inhibited at 12.5 *μ*g/ml of miconazole, which was the MIC obtained in the susceptibility tests. This finding also corresponds with the findings by other authors [38]. Bhattacharya et al. [39] reported the MIC of miconazole to be within the range of 0.2 *μ*g/ml to 25 *μ*g/ml against 20 *C. albicans* strains.

**Figure 12:**
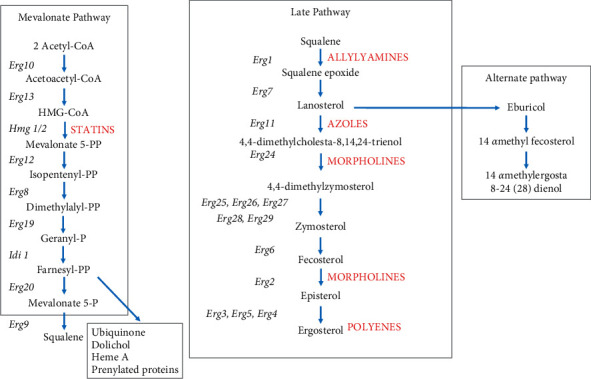
The ergosterol biosynthetic pathway. The pathway has been divided into three subpathways, the mevalonate pathway, the late pathway, and the alternative pathway, which can occur in the event of inactivation of lanosterol metabolizing enzymes. Shown in red are some common types of antifungal drugs at their site of action within this pathway adopted from [41].

The decrease in extracted ergosterol with increasing tormentic acid and DCM: methanol extract concentration may suggest that these *C. citrinus* phytochemicals impair the synthesis of ergosterol [40]. Available pharmaceutical drugs also use inhibition of ergosterol biosynthesis as a therapeutic mode of action (Figure 12). Azole drugs, in particular, inhibit the synthesis of ergosterol by binding to CYP 51 enzyme responsible for the demethylation of its sterol substrate, lanosterol [42]. The enzyme structure of CYP 51 is highly conserved in fungal cells, and mainly the heme iron within its porphyrin ring as well as amino acid sequences within the enzyme's active site governs its catalytic activity. Tyrosine residues and two weak hydrogen bonds hold the heme uniquely. His 310 and Asp 226 in the I and F helix, respectively, are involved in proton delivery. Within the I helix of the CYP fold, Thr 311 also plays an important role in the binding of substrates and inhibitors close to the iron group [43].

The higher antifungal potency of the DCM: methanol extract as compared to tormentic acid could be attributed to the various bioactive phytochemicals within this crude extract. The crude extract could contain one or more secondary metabolites that have antifungal activity.

The combinations of phytochemicals within this crude extract including tormentic acid may have additive or synergic effects on the impairment of ergosterol biosynthesis in *C. albicans* [31]. It is also possible that some of the phytochemicals within the crude extract have much higher antifungal activity, but the presence of tormentic acid and other phytoconstituents leads to an antagonistic effect where antifungal potency is reduced. Researchers have been able to isolate some biomolecules from *C. citrinus* that showed higher antifungal activity. The findings by Dakole et al. [44] show that essential oils of *C. citrinus* have fungicidal activity, with recorded MIC values of less than or equal to 6.25 *μ*g/ml. Rao et al. [45] found that isolated terpene, carvacrol, possessed antifungal activity, and the MIC of carvacrol was found to be 79.8 *μ*g/ml. Another study on terpene antifungal activity has also suggested the synergic activity of terpene within plant extracts [46]. Although isolation and characterization of phytochemical content were not carried out in this study, based on studies by other researchers, secondary metabolites present in the DCM: methanol extract would have caused the observed effects [14, 47].

Lanosterol metabolism involves the oxidation of the 14*α*-methyl group of lanosterol and subsequent demethylation of this group. The resemblance of tormentic acid structure to the conventional substrate of CYP51, lanosterol, may suggest similar interactions with this enzyme. Tormentic may be acting as a competitive inhibitor of CYP 51, thereby, reducing the activity of the enzyme on lanosterol per unit time. As tormentic acid also possesses a 14*α*-methyl carbon, it is possible that the enzyme competitively interacts with tormentic acid through demethylation [48].

It has also been reported that terpenes possess antifungal properties. These studies seem to suggest that the hydroxyl group and aromatic groups are important for antifungal activity [45]. Terpenes have also been shown to bind to CYP 51 by type 1 spectral binding which mimics the binding of lanosterol to CYP 51. Miconazole was able to completely inhibit ergosterol synthesis at 25 *μ*g/ml. This high potency maybe because miconazole, as with other azole drugs, binds directly to the heme group of CYP 51, thereby effectively inhibiting the action of this enzyme [43].

Ergosterol is present in cells in the form of free ergosterol, which carries out important functions in the cell membrane, as well as its esterified storage form. The esterified form of ergosterol is present in the cytosol [49]. In order, therefore, to extract all ergosterol synthesized by fungal cells, an initial saponification step was employed. Saponification was carried out by treating fungal pellets with alcoholic KOH and subjecting these cells to a high temperature of 80°C [50]. In so doing, fatty acid conjugates of ergosterol, as well as other lipids, such as phospholipids and triglycerides, are broken down [51].

For the extraction of ergosterol, *n*-hexane was used as a solvent as it has been proven to be a suitable solvent for sterol extraction [52]. This extraction solvent owes its sterol extracting capacity to its nonpolar nature and is recommended due to its low latent heat of vaporisation [53]. The solvent extracts ergosterol along with other nonpolar substances from the cells. These give the difference absorbance values obtained. Due to differences in chemical structures, different components within the samples have different absorption properties at different wavelengths within the electromagnetic spectrum. An intense spectrum for ergosterol, together with dihydroergosterol, is observed at 230 nm. Unfortunately, no independent peak for ergosterol on its own has been observed in previous studies. The quantification of ergosterol, therefore, was calculated as the difference in absorbance at 230 nm and that at 281.5 nm, where dihydroergosterol independently gives maximum absorbance [22].

## 5. Conclusion

*C. albicans* was more susceptible to tormentic acid and extracts of *C. citrinus* as compared to *C. tropicalis.* Extracts had more cytotoxic effects against *Candida* cells than tormentic acid. The DCM: methanol extract had the highest inhibitory potency against *Candida* cells. Tormentic acid significantly reduced the levels of ergosterol in *C. albicans*. The DCM: methanol extract reduced ergosterol significantly compared to tormentic acid. This may suggest synergistic effects of tormentic acid and other active compounds within the crude extract. Tormentic acid, therefore, has inhibitory effects against *Candida* cells, and the mode of action used by tormentic acid is ergosterol biosynthesis inhibition. A work published previously [54] reported the inhibition of protease production in *Staphylococcus aureus* by extracts from *C*. *citrinus* and tormentic acid. Thus, the current findings add on to the board of knowledge that tormentic acid and extracts from *C*. *citrinus* may be exploited for the development of antifungal agents, which also have antibacterial agents.

## Data Availability

The datasets generated during and/analysed during the current study are available from the corresponding author on reasonable request.

## References

[1] Sardi J. C. O., Scorzoni L., Bernardi T., Fusco-Almeida A. M., Mendes Giannini M. J. S. (2013). *Candida* species: current epidemiology, pathogenicity, biofilm formation, natural antifungal products and new therapeutic options. *Journal of Medical Microbiology*.

[2] Patil S., Rao R. S., Majumdar B., Anil S. (2015). Clinical appearance of oral *Candida* infection and therapeutic strategies. *Frontiers in Microbiology*.

[3] Calderone R. A., Fonzi W. A. (2001). Virulence factors of *Candida albicans*. *Trends in Microbiology*.

[4] Pfaller M. A., Diekema D. J. (2004). Rare and emerging opportunistic fungal pathogens: concern for resistance beyond *Candida albicans* and *Aspergillus fumigatus*. *Journal of Clinical Microbiology*.

[5] Singh S., Fatima Z., Hameed S. (2015). Predisposing factors endorsing *Candida* infections. *Le Infezioni in Medicina*.

[6] Höfs S., Mogavero S., Hube B. (2016). Interaction of *Candida albicans* with host cells: virulence factors, host defense, escape strategies, and the microbiota. *Journal of Microbiology*.

[7] Whaley S. G., Berkow E. L., Rybak J. M., Nishimoto A. T., Barker K. S., Rogers P. D. (2017). Azole antifungal resistance in *Candida albicans* and emerging non-albicans *Candida* Species. *Frontiers in Microbiology*.

[8] George B., Dhivya R. (2019). Phytochemical screening and antifungal activity of solvent extracts of *Averrhoa bilimbi* leaves against *Aspergillus niger* and *Rhizopus stolonifer*. *International Journal of Science and Healthcare Research*.

[9] Van De Bossche H., Willemsens G., Cools W., Lauwers W. F. J., Jeune L. L. (1978). Biochemical effects of miconazole on fungi and inhibition of ergosterol biosynthesis in *Candida albicans*. *Comparative Biochemistry and Pharmaceutical Interactions*.

[10] Hu Z., He B., Ma L., Sun Y., Niu Y., Zeng B. (2017). Recent advances in ergosterol biosynthesis and regulation mechanisms in *Saccharomyces cerevisiae*. *Indian Journal of Microbiology*.

[11] Esser K., Bennett J. W. (2002). *The Mycota: A Comprehensive Treatise on Fungi as Experimental Systems for Basic and Applied Research, Human Fungal Pathogens*.

[12] Aguilar P. S., Heiman M. G., Walther T. C. (2010). Structure of sterol aliphatic chains affects yeast cell shape and cell fusion during mating. *Proceedings of the National Academy of Sciences*.

[13] Ksouri S., Djebir S., Bentorki A. A., Gouri A., Hadef Y., Benakhla A. (2017). Antifungal activity of essential oils extract from *Origanum floribundum* Munby, *Rosmarinus officinalis* L. and *Thymus ciliatus* Desf. against *Candida albicans* isolated from bovine clinical mastitis. *Journal de Mycologie Médicale*.

[14] Ahhmed A., Ceylan D., Muchenje V. Phytochemicals in *Callistemon citrinus*: a novel therapeutic approach.

[15] Jazet P., Tatsadjieu L. N., Ndongson B. D., Kuate J., Paul H. (2009). Correlation between chemical composition and antifungal properties of essential oils of *Callistemon rigidus* and *Callistemon citrinus* of Cameroon against *Phaeoramularia angolensis*. *Journal of Medicinal Plants Research*.

[16] Kumar D., Sukapaka M., Babu G. D. K., Padwad Y. (2015). Chemical composition and *in vitro* cytotoxicity of essential oils from leaves and flowers of *Callistemon citrinus* from Western Himalayas. *PLoS One*.

[17] Alam S., Khan F. (2018). QSAR, docking, ADMET, and system pharmacology studies on tormentic acid derivatives for anticancer activity. *Journal of Biomolecular Structure and Dynamics*.

[18] Dimitrova L., Zaharieva M. M., Popova M. (2017). Antimicrobial and antioxidant potential of different solvent extracts of the medicinal plant *Geum urbanum* L. *Chemistry Central Journal*.

[19] Zapata A., Ramirez-Arcos S. (2015). A comparative study of McFarland turbidity standards and the densimat photometer to determine bacterial cell density. *Current Microbiology*.

[20] Wiegand I., Hilpert K., Hancock R. E. W. (2008). Agar and broth dilution methods to determine the minimal inhibitory concentration (MIC) of antimicrobial substances. *Nature Protocols*.

[21] Arun T., Rabeeth M. (2010). Genotoxic effect of paracetamol containing tablets in cultured human lymphocytes. *International Journal of Biomedical Research*.

[22] Arthington-Skaggs B. A., Jradi H., Desai T., Morrison C. J. (1999). Quantitation of ergosterol content: novel method for determination of fluconazole susceptibility of *Candida albicans*. *Journal of Clinical Microbiology*.

[23] Lee D., Jung L., Park J., Yoo K., Chung I. (2010). Cytotoxic triterpernoids from *Cornus kousa* fruits. *Chemistry of Natural Products*.

[24] Woo K. W., Han J. Y., Choi S. U., Kim K. H., Lee K. R. (2014). Triterpenes from *Perilla frutescens* var *acuta* and their cytotoxic activity. *Natural Products Sciences*.

[25] Chipenzi T., Baloyi G., Mudondo T., Sithole S., Fru Chi G., Mukanganyama S. (2020). An evaluation of the antibacterial properties of tormentic acid congener and extracts from *Callistemon viminalis* on selected ESKAPE pathogens and effects on biofilm formation. *Advances in Pharmacological and Pharmaceutical Sciences*.

[26] Krishna K. V. V. S., Surendra G., Anjana M., Siva Nagini K. S. K. (2012). Phytochemical screening and antimicrobial activity of *Callistemon citrinus* (L.) leaves extracts. *International Journal of PharmTech Research*.

[27] Fasola T. R., Iyamah P. C. (2014). Comparing the phytochemical composition of some plant parts commonly used in the treatment of malaria. *International Journal of Pure and Applied Sciences and Technology*.

[28] Iloki-Assanga S. B., Lewis-Luján L. M., Lara-Espinoza C. L. (2015). Solvent effects on phytochemical constituent profiles and antioxidant activities, using four different extraction formulations for analysis of *Bucida buceras* L. and *Phoradendron californicum*, complementary and alternative medicine. *BMC Research Notes*.

[29] Ahmad A., Khan A., Akhtar F. (2011). Fungicidal activity of thymol and carvacrol by disrupting ergosterol biosynthesis and membrane integrity against *Candida*. *European Journal of Clinical Microbiology & Infectious Diseases*.

[30] Burt S. (2004). Essential oils: their antibacterial properties and potential applications in foods-a review. *International Journal of Food Microbiology*.

[31] Petchayo S., Nguefack J., Yamdeu G., Hubert J., Fouelefack F. R. (2013). Antifungal potential of extracts from four plants against *Acremonium apii* and *Colletotrichum dematium*, two major pathogens of celery (*Apium graveolens* L.) in Cameroon. *International Journal of Current Science*.

[32] Srivastava S. K., Ahmad A., Jain N., Aggarwal K. K., Syamasunder K. V. (2001). Essential oil composition of *Callistemon citrinus* leaves from the lower region of Himalayas. *Journal of Essential Oil Research*.

[33] Salem M. Z. M., EL-Hefny M., Nasser R. A., Ali H. M., El-Shanhorey N. A., Elansary H. O. (2017). Medicinal and biological values of *Callistemon viminalis* extracts: history, current situation and prospects. *Asian Pacific Journal of Tropical Medicine*.

[34] Kim H.-M., Kwon H., Kim K., Lee S.-E. (2018). Antifungal and antiaflatoxigenic activities of 1,8-cineole and t-cinnamaldehyde on *Aspergillus flavus*. *Applied Sciences*.

[35] Cock I. (2012). Antimicrobial activity of *Callistemon citrinus* and *Callistemon salignus* methanolic extracts. *Pharmacognosy Communications*.

[36] Forastiero A., Mesa-Arango A. C., Alastruey-Izquierdo A. (2013). *Candida tropicalis* antifungal cross-resistance is related to different azole target (Erg11p) modifications. *Antimicrobial Agents and Chemotherapy*.

[37] Jin L., Cao Z., Wang Q. (2018). MDR1 overexpression combined with ERG11 mutations induce high-level fluconazole resistance in *Candida tropicalis* clinical isolates. *BMC Infectious Diseases*.

[38] Vandenbosch D., Braeckmans K., Nelis H. J., Coenye T. (2010). Fungicidal activity of miconazole against *Candida* spp. biofilms. *Journal of Antimicrobial Chemotherapy*.

[39] Bhattacharya S., Esquivel B. D., White T. C. (2018). Overexpression or deletion of ergosterol biosynthesis genes alters doubling time, response to stress agents, and drug susceptibility in *Saccharomyces cerevisiae*. *American Society of Microbiology*.

[40] Isham N., Ghannoum M. A. (2010). Antifungal activity of miconazole against recent *Candida* strains. *Mycoses*.

[41] Jordá T., Puig S. (2020). Regulation of ergosterol biosynthesis in *Saccharomyces cerevisiae*. *Genes*.

[42] Shareef M. A., Sirisha K., Khan I. (2019). Design, synthesis, and antimicrobial evaluation of 1,4-dihydroindeno[1,2-c]pyrazole tethered carbohydrazide hybrids: exploring theirin silicoADMET, ergosterol inhibition and ROS inducing potential. *MedChemComm*.

[43] Hargrove T. Y., Friggeri L., Wawrzak Z. (2017). Structural analyses of *Candida albicans* sterol 14*α*-demethylase complexed with azole drugs address the molecular basis of azole-mediated inhibition of fungal sterol biosynthesis. *Journal of Biological Chemistry*.

[44] Dakole D., Nguefack J., Dongmo L. J. B., Galani Y. J. H., Aza U. R., Amvam Z. P. H. (2016). Antifungal potential of essential oils , aqueous and ethanol extracts of thirteen plants against *Fusarium oxysporum* f. sp *Lycopersici* and *Phytophtora infestans* (Mont.) de Bary as major tomato pathogens in Cameroon. *International Journal of Current Science*.

[45] Rao A., Zhang Y., Muend S., Rao R. (2010). Mechanism of antifungal activity of terpenoid phenols resembles calcium stress and inhibition of the TOR pathway. *Antimicrobial Agents and Chemotherapy*.

[46] Pemmaraju S. C., Pruthi P. A., Prasad R., Pruthi V. (2013). *Candida albicans* biofilm inhibition by synergistic action of terpenes and fluconazole. *Indian Journal of Experimental Biology*.

[47] Sales T. A., Cardoso M. D. G., Guimarães L. G. D. L. (2017). Essential oils from the leaves and flowers of *Callistemon viminalis*: chemical characterization and evaluation of the insecticide and antifungal activities. *American Journal of Plant Sciences*.

[48] Hu C., Zhou M., Wang W., Sun X., Yarden O. (2018). Abnormal ergosterol biosynthesis activates transcriptional responses to antifungal azoles. *Frontiers in Microbiology*.

[49] Yuan H., Ma Q., Ye L., Piao G. (2016). The traditional medicine and modern medicine from natural products. *Molecules*.

[50] Hansen H., Wang T. (2015). Does the saponification-GC method underestimate total cholesterol content in samples having considerable cholesterol esters?. *Journal of the American Oil Chemists’ Society*.

[51] McDonald J. G., Smith D. D., Stiles A. R., Russell D. W. (2012). A comprehensive method for extraction and quantitative analysis of sterols and secosteroids from human plasma. *Journal of Lipid Research*.

[52] Nwabueze T. U., Okocha K. S. (2008). Extraction performances of polar and non-polar solvents on the physical and chemical indices of African breadfruit (*Treculia africana*) seed oil. *African Journal of Food Science*.

[53] Kumar S. P. J., Prasad S. R., Banerjee R., Agarwal D. K., Kulkarni K. S., Ramesh K. V. (2017). Green solvents and technologies for oil extraction from oilseeds. *Chemistry Central Journal*.

[54] Mashezha R., Mombeshora M., Mukanganyama S. (2020). Effects of tormentic acid and the extracts from *Callistemon citrinus* on the production of extracellular proteases by *Staphylococcus aureus*. *Biochemistry Research International*.

